# Discovering favorable genes, QTLs, and genotypes as a genetic resource for sesame (*Sesamum indicum* L.) improvement

**DOI:** 10.3389/fgene.2022.1002182

**Published:** 2022-11-01

**Authors:** Habtamu Kefale, Linhai Wang

**Affiliations:** ^1^ Key Laboratory of Biology and Genetic Improvement of Oil Crops, Ministry of Agriculture, Oil Crops Research Institute, Chinese Academy of Agricultural Sciences, Wuhan, China; ^2^ Department of Plant Science, College of Agriculture and Natural Resources, Debre Markos University, Debre Markos, Ethiopia

**Keywords:** candidate gene, genetic resource, genotype, linkage map, QTL, sesame

## Abstract

Sesame (*Sesamum indicum* L.) is an ancient diploid oilseed crop with high oil content, quality protein, and antioxidant characteristics that is produced in many countries worldwide. The genes, QTLs, and genetic resources of sesame are utilized by sesame researchers and growers. Researchers have identified the many useful traits of this crop, which are available on different platforms. The genes, genotypes, QTLs, and other genetic diversity data of sesame have been collected and stored in more than nine genomic resources, and five sesame crop marker databases are available online. However, data on phenotypic and genotypic variability, which would contribute to sesame improvements, are limited and not yet accessible. The present study comprehensively reviewed more than 110 original published research papers and scientifically incorporated the results. The candidate genes, genotypes, and QTLs of significantly important traits of sesame were identified. Genetic resources related to grain yield and yield component traits, oil content and quality, drought tolerance, salt tolerance, waterlogging resistance, disease resistance, mineral nutrient, capsule shattering resistance, and other agronomic important traits of sesame were studied. Numerous candidate genotypes, genes, QTLs, and alleles associated with those traits were summarized and discovered. The chromosome regions and linkage groups, maps associated with the best traits, and candidate genes were also included. The variability presented in this paper combined with sesame genetic information will help inform further sesame improvement.

## Background

Sesame (*Sesamum indicum* L., 2*n* = 26), an ancient oilseed crop that belongs to the Pedaliaceae family, is widely cultivated in Africa and Asia (Zhang et al., 2013b; Stavridou et al., 2021; Wang et al., 2021). Although the origin and domestication are controversial, Ethiopia (Dagmawi et al., 2015; Boru, 2021; Teklu et al., 2021), India (Bedigian and Harlan, 1986; Bedigian and Korosec-Koruza, 2003), Sudan (Bedigian and Korosec-Koruza, 2003), and China (Nayar and Mehra, 1970) are believed to be the sources of sesame origin and domestication. Sesame includes more than 34 species with different chromosome numbers and features (Nayar and Mehra, 1970). Sesame global production has reached 11.7 million hectares (Mha) and 6.02 MT production, with an annual average productivity of 0.52 ton ha^−1^ (Yadav et al., 2022). The leading sesame-producing countries in 2020 were Sudan (1.53million MT), Myanmar (740, 000 MT), Tanzania (710, 000 MT), India (658,000 MT), Nigeria (490, 000 MT), China (447,000 MT), Burkina Faso (270, 000 MT), and Ethiopia (260, 000 MT) (https://www.tridge.com/production?code=0289&producer=WL).

Sesame seeds are recognized for their high-quality oil, high protein content, vitamins, and unique antioxidant compounds like sesamin and sesamolin, which contribute to sesames’ popularity as a healthy and nutritious food (Liang et al., 2021). Sesame has excellent characteristics, including a large propagation coefficient (3,000–10,000 seeds/plant), relatively short growing season (3 months), high drought resistance, high oil content (55%), and small diploid genome (∼337 MB [Wang et al., 2014] to ∼350 MB [Wei et al., 2017]). Sesame seeds are mostly used for cooking oil; the flour left over after oil extractions contains 35–50% protein and is utilized as feed for cattle and poultry (Tripathy et al., 2019; World Atlas). Sesame has not only edible oil (dietary) but also compounds that could be used for therapeutics in medicine and cosmetics applications (Tripathy et al., 2019).

Around 35,000 sesame accessions have been provided worldwide, from which researchers have identified the allelic, gene, and genomic variations of sesame populations (Wei et al., 2017). Superior alleles and genes for different sesame traits can be found in germplasm resources preserved in numerous national and international sesame gene banks. These resources are a source of candidate genes, genotypes, and agronomically important traits for sesame research and improvement programs. Candidate genes and QTLs associated with oil content and quality can be discovered by GWAS, which provides precise clues for uncovering the genetic mechanism for important agronomic traits of sesame (Wei et al., 2017). Although summarized sesame population diversity information is still not available (Satyendra Nath et al., 2014), the location of sesame origin indicates the place of diversity and source of rich gene and genetic resources, which is true for any crop species.

Agro-morphological studies have reported the genetic diversity of sesame genetic resources. In recent years, molecular-level or PCR-based methods of genetic diversity have also played vital roles in developing gene resources and demonstrating the diversity of the sesame population. However, the genetic diversity between sesame cultivars is reportedly lower due to the source of variability originating from one formerly cultivated sesame species (Zhang et al., 2012). Although the genetic diversity of sesame is limited to one cultivated species, the rich phenotypic and genotypic diversity within the species indicates the presence of valuable functional gene resources for genomic studies to improve sesame. Therefore, this comprehensive review highlights the available genotype and gene resources of sesame from more than 110 published journal articles.

## Sesame germplasms and diversity

The presence of genetic diversity and variability between and within the species is the first criterion for any crop improvement. Most of the variability, diversity, and characterization studies of sesame have aimed to identify candidate genotypes and genetic resources to improve sesame traits. *Sesamum indicum* L. is available in different countries and continents worldwide for its multipurpose high-quality oil from seeds, as well as other byproducts (Teklu et al., 2021). Sesame has more than 35,000 accessions in international major crop germplasm gene banks (Wei et al., 2017). In their review, Dossa et al. (2017a) reported that approximately 26,000 sesame genetic materials are conserved in four principal international gene banks, including the Indian-NBPGR-national gene bank, South Korea [National Agro-Biodiversity Center (NABC) and Rural Development Administration (RDA)], China-Oil Crops Research Institute CAAS, and the United States (United States Department of Agriculture [USDA], Agricultural Research Service [ARS], and Plant Genetic Resources Conservation Unit [PGRU]). Korea and the US (USDA, ARS, and PGRU) have preserved 7,698 (He et al., 2019) and 1,226 (Morris, 2009) sesame accessions respectively. Recently, more than 8,000 accessions in China (Berhe et al., 2021) and 6,658 sesame accessions in India NBPGR (Mahajan et al., 2007) have been reported. In this regard, Dagmawi et al. (2015) indicated that around 870 sesame accessions were collected and preserved at the Ethiopia Biodiversity Institute (EBI). Dossa et al. (2017b) also reported unknown preserved sesame genetic resources in Nigeria, Ethiopia, and Sudan. However, information is insufficient regarding the sesame genetic resources in each major germplasm seed bank worldwide. Therefore, it is important to establish online databases to record all genetic resources globally to reduce the double counting of sesame materials.

The main goals of sesame breeding are to improve seed yield and oil quality. Achieving this breeding objective requires gene and genetic diversity. The first step and major task for a plant breeder is to identify the potential genes controlling essential traits of a given crop (Pham et al., 2011). Genetic diversity can be achieved using landraces and wild species in conventional breeding as sources of valuable traits for wider adaptability, including traits related to biotic and abiotic stress resistance (Nyongesa et al., 2013; Teklu et al., 2022). Sesame includes more than 37 species distributed across Africa, Asia, and Australia, with different chromosome numbers and features (Nayar and Mehra, 1970; Yadav et al., 2022). The ploidy level of *S. indicum* and *S. radiatum* are expressed as 2*n* = 26 and 2*n* = 64, respectively (Nayar and Mehra, 1970). The somatic chromosome numbers of some wild species of sesame include *S. lanciniatum* at 2*n = 28* and *S. angolase* and *S. prostratum*, at 2*n* = 32, and *S. occidentale*, at 2*n* = 64 (Hegde, 2012). Other species include *S. triphyllum*, and *S. capense*, with 2*n* = 26, *S. angustifolium*, with 2n = 32, and *S. prostratum* and *S. schinzianum*, with 2*n* = 64 (Yadav et al., 2022). Therefore, sesame has species with various ploidy levels, including diploid (*S. indicum*, *S. triphyllum*, and *S. capense*, where 2*n* = 2*x* = 26), tetraploid (*S. angustifolium*, *S. angolase*, and *S. prostratum*, where 2*n* = 32), and octaploid (*S. occidentale*, *S. radiatum*, and *S. schinzianum*, where 2*n* = 8*x* = 64).

Nyongesa et al. (2014) studied the genetic relationships among a total of 46 accessions, including 37 cultivated *S. indicum* accessions and 11 accessions from wild species of *S. angolense*, *S. spp.*, *S. latifolium*, and *S. calycinum* based on somatic chromosome counts and isozyme markers. They observed consistent numbers of somatic chromosomes (2*n* = 32) from the four wild species, unlike those in the cultivated species (2*n* = 26), indicating the presence of genetic variation and diversity. Therefore, the utilization of the wild relatives of sesame is critical for increasing genetic diversity and mining important genes associated with traits to react to biotic and abiotic stress. Kumari and Ganesamurthy (2015) performed direct and reciprocal crosses between wild sesame species including *S. alatum*, *S. malabaricum*, *S. radiatum*, and a wild variety of *S. indicum* (*S. indicum* var. *yanamalaiensis*) and other cultivated variety of *S. indicum*. They observed significant variations between cultivated and wild species of sesame in various traits including leaf pubescence; flower size; corolla and anther color; capsule size and shape; color; extra floral nectary size and shape; and seed size, texture, and branching pattern. The cross-compatibility analyses revealed that *S. indicum* is compatible with *S. malbarum* and incompatible with *S. alatum* and *S. radiatum* and partially compatible with *S. indicum var. yanamaliansis* in direct and reciprocal crosses (Kumari and Ganesamurthy (2015). Additionally, the authors recommended the wild species *S. malabaricum* and *S. indicum* var. *yanamalaiensis* for the transfer of essential traits from wild to cultivated sesame species*.*

Genetic diversity can be studied using three main methods: agro-morphological, biochemical, and molecular marker methods. Studies on the gene/genetic diversity and divergence of crop species, candidate genes, QTLs, and genotypes have been reported worldwide. The candidate genes and genotypes provide genetic resources for crop improvement programs. Tripathy et al. (2019) reported more than 270 genotypes developed for different agronomic traits showing the best performance in some sesame-growing countries. Grain yield, disease resistance, drought tolerance, oil quality, coat color of seed and flower, plant height, capsule number, seed per capsule, capsule length, shattering resistance, oil content, and leaf morphology are the most important traits for sesame improvement (Pham et al., 2011; Sheng et al., 2021; Stavridou et al., 2021).

The phenotypic variability of the sesame genotypes directly indicates the agro-morphological traits and indirectly suggests the genes and genetic variability controlling them. Teklu et al. (2021) reported 10 genotypes with superior phenotypic (grain yield and oil yield) and genotypic variability from among 100 populations, including Hirhir Humera Sel-6, Setit-3, Hirhir Kebabo Hairless Sel-4, Hirhir Nigara 1st Sel-1, Humera-1, and Hirhir Kebabo Early Sel-1 (from cluster II-a), Hirhir kebabo hairless-9, NN-0029 (2), NN0068-2, and Bawnji Fiyel Kolet (from cluster II-b). The authors observed 30–25 polymorphisms and gene diversity index values by using SSR molecular markers in these populations. Their findings demonstrated the genetic variability and diversity in the studied sesame genetic resources, which suggested the genetic potential of these materials. Therefore, such genetic resources provide important candidate genotypes and can be used for further sesame improvement programs.

A study of genetic diversity assessed Mediterranean core collections including 95 accessions and 21 geographic regions spread over four different continents of Africa, Asia, America, and Europe (Basak et al., 2019). The authors reported the highest allelic variations in accessions collected from Asia compared to those from Africa, America, and Europe, suggesting that the Mediterranean core collections are highly genetically diverse. Similarly, Wei et al. (2015) reported abundant phenotypic diversity and 56 agronomical important traits for sesame improvement from among 705 accessions in their diversity analysis study conducted in China. Consequently, Adu-gyamfi et al. (2019) observed genetic diversity among the 25 accessions in Ghana based on the analysis of morphological and molecular data.

The most important traits assessed by genetic diversity studies include oil content and quality, vitamin and nutrient composition, yield and yield component traits, morphological characteristics, growth cycle, seed color, and disease resistance (Dossa et al., 2017a; Basak et al., 2019; Teklu et al., 2022). A study of genomic variations between the cultivated variety “Zhonghzi13” and two landraces (Baizhima and Mishuozhima) in China observed more differences in important agronomic traits such as plant height, branches, seed size, seed number per capsule, disease resistance, and flowering date (Wei et al., 2016). A large-scale GWAS study reported a total of 169 sets of phenotypic data based on 1,805,413 standard SNPs with low allelic frequency (MAF) > 0.03 (Wei et al., 2015). Likewise, from a total of 120 (82 Ethiopian and 38 exotic) accessions, increased polymorphism was observed in the Ethiopian accession collections (75.85) compared to that in the exotic accession collections (65.52) based on six ISSR primers (Dagmawi et al., 2015). The accessions with higher polymorphism indicate the presence of important alleles and genes controlling traits not found in other accessions (exotics). Therefore, the Ethiopian collections could be valuable genetic resources for sesame improvement.

Wang et al. (2014) reported that the resequencing of 29 sesame accessions from 12 different countries revealed higher genetic diversity in lipid-related genes, suggesting an association with wide variation in oil content. Hernán. (2007) observed higher variability within the diversity centers. Kehie et al. (2020) also reported considerable variations in all traits included in their analysis among 25 sesame genotypes. The variability of sesame indicates the opportunities for developing and improving sesame genotypes for better agronomic, stress tolerance, and other quality traits.

Studies on genetic diversity have applied different classical and molecular markers, including morphological (agronomical important traits of sesame, oil content, yield, plant height, number of capsules/plant, seed/plant, seed coat color) and PCR-based (RFLP, SSR, SNPs, etc.) (Dagmawi et al., 2015; Basak et al., 2019) methods. The variations in different traits, ranging from allelic to phenotypic, indicate the presence of candidate gene resources and the potential for variability that could be used in sesame breeding programs. Accessions of landraces and wild types are a source of valuable traits and should be maintained and conserved for future sesame improvement programs.

## Sesame genomes and databases

The reported genome size of sesame varies from 369 Mb (Zhang et al., 2013a), to 357 Mb (Wang et al., 2014), 350 Mb (Wei et al., 2017), whereas and ∼375 Mb (Sabag et al., 2021). Thus, the average genome size is ∼360 Mb, which is not larger than those of other oil crop species, including sunflower ∼3500 Mb (Staton et al., 2012), soybean ∼1,013.2 Mb (Xie et al., 2019), and rapeseed ∼1200–1280 Mb (Song et al., 2020). The variation in genome size might be due to the differences in the sequenced genotype, sequencing methods, and the presence of transposon elements. Sinbase 1.0 (http://orci-genomics.org/Sinbase/) was the first version of a database used as a reference for genomic and bioinformatics analysis, which was launched 7 years ago by the Oil Crops Research Institute Chinese Academy of Agricultural Sciences (CAAS) (Wang et al., 2015). This is the world’s first comprehensive and integrated resource platform for sesame genetics, genomes, and comparative genomics, and provides a user-friendly interface for quick access to sesame genome data. Sinbase 1.0 includes a total of 401,063 entries for genomic information, including 27,148 predicted protein-coding genes, 372,167 jumping genes, and 1,748 non-coding RNAs with 16 linkage groups. It also includes 406 genetic markers including single nucleotide polymorphism (SNPs), simple sequence repeats (SSRs), and insertion/deletion (indel) genetic markers and 16,296 scaffolds (Wang et al., 2015).

**FIGURE 1:**
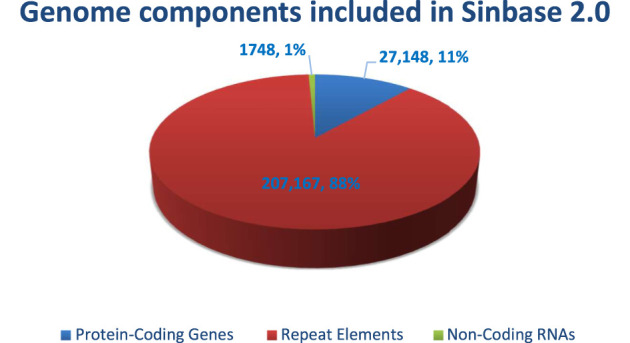
Genome components integrated in Sinbase 2.0.

Sinbase 1.0 was recently upgraded to version 2.0, which includes updated genome sequence data (Wang et al., 2021). This advanced, updated, and multi-omics Sinbase database is an online, freely downloaded, and accessible gene resource for sesame improvement (http://www.sesame-bioinfo.org/Sinbase2.0) that provides an updated genome database with 13 chromosomes, three genetic linkage maps, five intra- and six inter-species genomic comparisons, one genomic variation analysis, data from five transcriptomes and one proteome, 31 functional markers, 175 putative functional genes and 54 QTLs from agronomic important traits (Wang et al., 2021). Updated genomics; genetics; comparative genomics; transcriptomes; proteomics; and functional markers, genes, and QTLs using multiple-layer methods according to different multi-omics data formats have also been integrated into Sinbase 2.0 (Wang et al., 2021). This internet-based database includes a total of 236,063 pieces of genome information including protein-coding genes (27,148), repeat elements (207, 167), and non-coding RNAs (1,748). In this database, the highest component of the genome which accounts for 88% was the protein-coding regions whereas non-coding RNAs comprise 1% (Figure 1).

In addition, since 2014, the new and novel online *Sesamum indicum* L. genetic discovery database (SiGeDiD) (https://sigedid.ucad.sn/) has been constructed to facilitate access to all genetic and genomic findings in sesame using GWAS (Berhe et al., 2021). Currently, more than nine online comprehensive databases of sesame related to gene expressions, QTLs, gen families, functional genes, comparative genomics, and phenotypes (Table 1), as well as five platforms focusing on sesame marker development and utilization (SSRs, InDels, SNPs, and AFLPs) transposons, genetic maps, and haplotype maps (Table 2). The availability of this genetic information through online platforms and other sources indicates the advancement of sesame research and will play a significant role in the breeding and crop improvement programs of sesame and other oil crops.

**TABLE 1:** Sesame genome online databases and numbers of integrated genomes, traits, QTLs, and markers.

No.	Name of sesame online database and their webpages	Number of functional QTLs and traits	Source genomes	Markers developed/identified	References
1	Sinbase 1.0 http://ocri.genomics.org/Sinbase/	An unknown number of QTLs but the traits are oil content, oil biosynthesis, sesamin, and sesamolin production, lipid-related genes	Zhonghzi13	2719 SNPs, 97InDels and 2,282 SSRs	Wang et al. (2014), Wang et al. (2015)
2	Sinbase2.0 http://www.sesame-bioinfo.org/Sinbase2	54 QTLs, The traits are disease resistance, growth cycle morphological features, yield component, oil quality, oil content, and abiotic stress resistance traits	Zhonghzi13	31 functional markers	Wang et al. (2021)
3	Genetic Discovery Database (SiGeDiD) (https://sigedid.ucad.sn/)	65 QTLs. The traits are seed coat color, capsule zone length, tip length without capsule, internode length, node number, plant height, the height of first capsule, grain number/capsule, capsule number/plant, waterlogging tolerance, and capsule axis	Unknown	34 functional markers	Berhe et al. (2021)
4	Sesame Functional Genomics database (SesameFG) http://www.ncgr.ac.cn/SesameFG	The traits included are yield, yield-related, disease resistance, oil quality, growth cycle related, flowering date, waterlogging, lipid metabolism, and other morphological traits	Zhonghzi13 and Baizhima	Genomic-104,836 SSR markers -Polymorphism SSRs-218	Wei et al. (2017)
5	Sesame HapMap http://202.127.18.228/SesameHapMap/or	549 QTLs, Totally 56 traits are included and some are oil content, fatty acid biosynthesis, nutritional quality, morphological feature traits, seed color, growth cycle, oil seed yield, disease resistance, and yield-related traits	705 accessions	A total of 5,407,981 SNPs	Wei et al. (2015)
6	The sesame genome project working group (SGPWG) http://www.sesamegenome.org	Not mentioned	Yuzhi-11	Not determined	Zhang et al. (2013a)
7	NCBI assembly database. https://www.ncbi.nlm.nih.gov/assembly/?term=Sesamum+indicum	Not accessible (need permission)	Yuzhi-11, Zhongzhi-13 and Swetha	Not accessible	Kitts et al. (2016)
8	Plant Tandem duplicated Genome database (PTGBase) http://ocri-genomics.org/PTGBase/	Not accessible (need permission)	Zhonghzi13	Not accessible (need permission)	Yu et al. (2015)
9	Sesame Pan-genome References database (SesamePan-genome) database. http://www.sesame-bioinfo.org/pan-genome/	Lipid metabolism (fatty acid elongation, cutin, suberin, and wax biosynthesis, steroid hormone biosynthesis, glycerolipid biosynthesis, glycerophospholipid metabolism, ether lipid metabolism, alpha linolic acid metabolism, sphingolipid metabolism, and unsaturated fatty acid biosynthesis)	Five genomes; Zhongzhi-13, Baizhima, Mishuozhima, Swetha and Yuzhi-11	Not accessible (need permission)	Yu et al. (2019)

**TABLE 2:** Online databases for sesame markers.

No.	Name of molecular markers database	Websites	Number of markers integrated	References
1	Sesame SNPBase	http://www.sesamebioinfo.org/SesameSNPresults	Not accessible	
2	Database for SSR analysis in *Sesamum indicum* L (Sisatbase)	http://www.sesamebioinfo.org/SisatBase/index.html	138,194 SSRs	Dossa et al. (2017a)
3	Plant Microsatellite DNAs Database(PMDBase)	http://www.sesamebioinfo.org/PMDBase/index.html	26, 230,099 SSRs	Yu et al. (2017)
4	Genome wide microsatellite marker database (GinMicroSatDb)	http://backwin.cabgrid.res.in:8080/Gingelly7/	Totally identified 118,004 SSRs but, 21, 704 SSRs were integrated	Purru et al. (2018)
5	Oil Crops Seed (ocsESTdb)	http://ocri-genomics.org/ocsESTdb/	2, 328, 925 EST for four different crops (sesame, rape seed, soybean, and peanut). However, Sesame has 44,820 ESTs	Ke et al. (2015)

To enhance sesame improvement projects, Dossa et al., 2016 reported on essential genomic sequence resources such as functional markers, genes, and QTLs associated with agronomically desired traits, which were created using linkage mapping and association analysis. Dossa also created a physical map of important QTLs, functional markers, and genes available for the sesame breeding program. These resources could be helpful for sesame research and provide an excellent view of genetic information. Berhe et al. (2021) recently reported around 300 QTLs and 250 functional genes related to the qualitative and quantitative traits of sesame.

**FIGURE 2:**
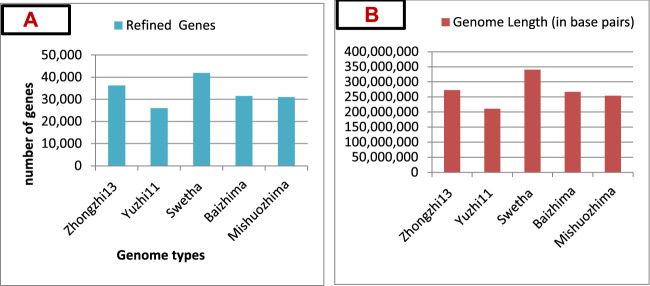
**(A)** Genomes sequenced and their refined number of genes. **(B)** Genome lengths (in base pairs) of five cultivars (genomes). Data source: Yu et al. (2019).

Regarding sesame genome database development, more than five sesame genotypes of cultivated and landraces have been sequenced and their genomic data assembled (Yu et al., 2019; Wang et al., 2021), including Zhongzhi-13 (Wang et al., 2014), Yuzhi-11 (Zhang et al., 2013a), Swetha (Kitts et al., 2016), Mishuozhima, and Baizhima (Wei et al., 2015). Based on the numbers of refined genes and genome length, Swetha showed a higher number of genes (41, 859) (Figure 2A) and genome length (340,463,922 base pairs) (Figure 2B) (Yu et al., 2019). Therefore, the Swetha Indian cultivar has a large genome size compared to other genotypes and could be a source of unique genes and QTL resources.

**FIGURE 3:**
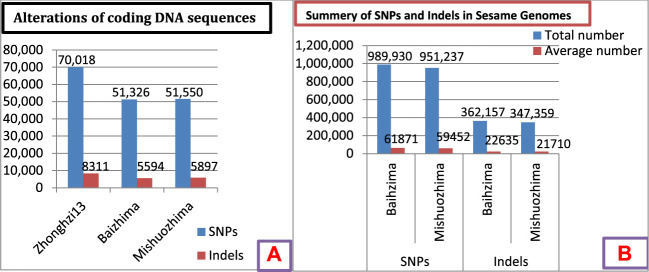
**(A)** Alterations of coding DNA sequences in three genomes. **(B)** Summary of SNPs and indels in two sesame cultivars. Data source: Wei et al. (2016).

Wei et al. (2016) observed large genomic variations in three sesame genomes (Figures 3A,B). The genome of cultivar Zhonghzi-13 showed higher numbers of SNPs and indels in the DNA coding region compared to Baizhima and Mishuozhima (Figure 3A). Similarly, the Baizhima cultivar showed higher numbers of SNPs and indels compared to Mishuozhima (Figure 3B). This indicated higher variability in these markers, indicating a larger genome size and potential for marker development. Therefore, the genomes showing the large size and higher alteration of coding DNA sequences should be considered an opportunity for further sesame research and improvement.

## Sesame markers and genetic linkage maps

Sesame improvement programs have developed and implemented many diverse markers. Molecular markers are important for evaluating genetic diversity, identifying candidate genes, producing QTLs, analyzing linkage maps, performing genotyping, and performing marker-assisted selection. Both classical and molecular markers have been utilized by sesame improvement programs (Dossa et al., 2017b). Dossa et al. (2017a) reported the development and application of around 7,000 validated and more than 100,000 non-validated microsatellites (SSR) molecular markers for sesame research and development programs. Random applied polymorphic DNA (RAPD) and amplified fragment length polymorphism (AFLP) markers were grouped in the first class whereas different SSR molecular markers were grouped in the 2nd class. From the first class of molecular markers, AFLP for genetic relationship and diversity (Laurentin & Karlovsky, 2006) and RAPD (Bhat, et al., 1999; Gulhan A et al., 2004; Abdellatef et al., 2008) markers were successfully used in sesame improvement. SSR (Nyongesa et al., 2013; Badri et al., 2014) ISSR (Dossa et al., 2016; Hota et al., 2016), EST-SSR (Wenliang et al., 2011; Yepuri et al., 2013; Wu et al., 2014), cDNA-SSR (Wang et al., 2012), SNPs (Satyendra Nath et al., 2014), and G-sequence SSR (gSSR) (Zhang et al., 2012) markers were developed and also used in the evaluation, characterization, and genotyping of sesame diversity.

Wei et al. (2008) discovered 155 EST-SSR markers and successfully amplified 108 loci by using 44 EST-SSR markers, with an average of 2.45 loci per primer. Similarly, in sesame transcriptomes, 7,702 EST-SSRs were identified as potential molecular markers, with an average frequency of 8.3% (Wei et al., 2011). Badri et al. (2014) also reported 120 SSRs of which 25 microsatellite markers were developed from a selective hybridization strategy and 95 expressed sequence tags were extracted from the NCBI database. Subsequently, Wu et al. (2014) developed 3,769 single nucleotide polymorphic (SNPs) markers, and 89 PCR-based polymorphic markers including 44 SSR, 10 gnomic-SSR, and 35 insertion-deletion (InDels) markers. Wang et al. (2014) reported 2,719 SNPs, 97 indels, and 2,282 SSR markers from the genotyping of Zhuongzi-13. Furthermore, Uncu et al. (2016) reported 15, 521 high-throughput SNP markers using the genotyping by sequencing (GBS) approach. Wang et al. (2017) also developed 7,357 SSRs from the sesame genome and transcriptomes from a cross-population of 548 recombinant inbred lines (RILs) to construct a genetic linkage map with 424 polymorphic markers. More recently, Cui et al. (2021) reported 224 significantly polymorphic SNP markers from 336 sesame germplasm lines in a GWAS study of seed coat color in 12 different environments. They also identified four markers (S1_6648896, S2_12232938, S7_6839839, and S8_8313501) as the most reliable and significant SNP markers.

Zhang et al. (2013c) reported 71, 793 SLAF markers for the constriction of sesame genetic maps. The authors also confirmed the importance of the SLAF-seq method in developing a large number of high-accuracy markers with less sequencing. Therefore, this method is important and especially suited for species with low polymorphism, like sesame. Kizil et al. (2020) reported 86 indel sites with long lengths (>8Mbs) in genome-wide analysis. They selected and used 16 of these markers to identify the diversity of 32 sesame accessions with an average of 0.33 polymorphisms in double-digest restriction site-associated DNA sequencing (ddRADSeq) data.

Transcriptome sequencing was used to identify 2,164 genic-SSR (gSSR) from sesame in China (Zhang et al., 2012). Consequently, Zhang et al. (2012) reported 59 cDNA SSR markers in 36 individuals showing allelic heterozygosities of 2-4 per locus. Yepuri et al. (2013) reported 16,619 EST-SSR from the gene bank of India (NCBI’s) database. From this database, 156 primer pairs were considered and characterized to determine the diversity of 49 sesame accessions. These markers increase the number of SSR marker resources and created an opportunity for increased genetic diversity, qualitative and quantitative trait mapping, and marker-assisted selection studies in sesame. The development of high-quality amplicons from many EST-SSR markers indicates the suitability of EST markers for specific primer designs (Wei et al., 2011). Moreover, the marker data of sesame may be used to improve molecular markers and sesame genetic information.

More than five online sesame molecular marker databases have been established for different marker types (Table 2). Yu et al. (2017) developed PMDBase (http://www.sesame-bioinfo.org/PMDBase) for SSR marker development and DNA research in plants, which includes around 26,230,099 microsatellites for 110 eukaryotic species, with different functions and software. This tool help inform breeding efforts, including the identification of genes responsible for seed coat color. Similarly, Purru et al. (2018) developed the GinMicrosatDb database (http://backwin.cabgrid.res.in:8080/Gingelly7/) of microsatellite (SSR) markers, including different options; i.e., the selection of markers, primers, flanking sequences, physical maps, and a genome browser. The 118,004 markers identified in the “Swetha” sesame variety are available in this online database. Although various marker databases have been established, the web pages of some are inaccessible, possibly due to security or maintenance issues. However, the information included in the present study was also obtained from the original studies.

**FIGURE 4:**
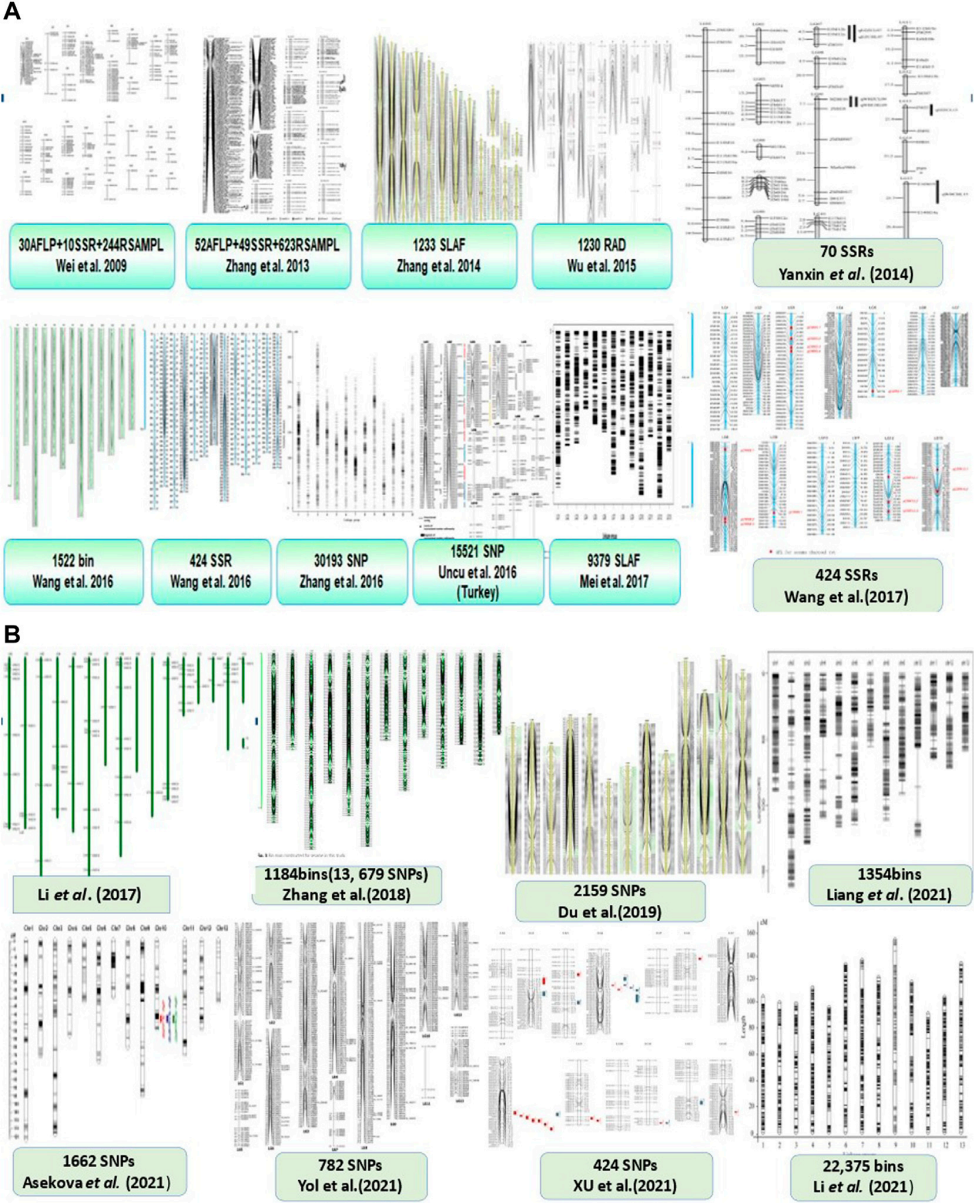
The status of Sesame Genetic linkage maps for different traits using different molecular markers. **(A)** First batch of linkage maps. **(B)** Recent linkage maps of sesame. More than 18 genetic linkage map collections on sesame from the past to the present are shown.

Genetic linkage mapping is an efficient method to show the specific location of genes and the distance between genes and markers on a chromosome (Wu et al., 2014). There are different tools used to map genes and QTLs on a chromosome including (GCIM, ICIM, IM, CIM (Li et al., 2021), with MCIM and MIM (Wu et al., 2014). Li et al. (2021) applied for the mapping of sesame seed coat color. More than 18 different genetic linkage maps for sesame different traits have been reported (Figure 4). The first sesame gene linkage map was constructed from the intraspecific cross results of F2 populations using 284 polymorphic PCR-based markers (Wei et al., 2009). However, Zhang et al. (2013c) constructed and reported the first high-density genetic map of sesame developed using a new marker type and the specific length amplified fragment sequencing (SLAF-seq) method. Yanxin et al. (2014) also constructed a genetic linkage map length of 592.4 cM using 70 polymorphic markers that clustered into 15 linkage groups (LG). The development of a high-quality genetic map would provide the best opportunity for genome assembly and the mapping of the quantitative trait loci/genes of agronomic traits of important oil crops including sesame (Wang et al., 2016a). Zhang et al. (2016) developed an ultrahigh genetic linkage map of *SiDT* genes in 13 LGs governing determinate growth habits of sesame using a total of 30,194 SNP markers.

Although Zhang et al. (2013c) reported 71,793 SLAF and 1,272 polymorphic markers for the construction of genetic linkage maps, only 1,233 markers were included on 15 LGs (chromosomes) with a 1.2 cM average distance between adjacent markers. This indicates the fine resolution, with very small marker distances to identify and isolate the genes of interest using SLAF markers. Therefore, the development of SLAF markers is an important platform for gene/QTL mapping, map-based gene identification, and isolation, as well as a reference for developing sequence scaffolds on physical maps, sesame genome sequence assembly, and other sesame molecular breeding programs.

Wu et al. (2014) reported the first high-density genetic linkage map of yield-related traits using the RIL population in sesame. Wu et al. (2014) also mapped 1,230 markers in 14 linkage groups, with a length of 844.46 cM and 0.69 cM average distance between adjacent markers. The authors applied RAD-Seq to identify 3,804 pairs of new and novel DNA markers including SNPs and indels to aid in the development of high-density genetic maps combined with SSR markers. Additionally, Wang et al. (2017) developed a genetic map with 13 linkage groups (chromosome numbers) and discovered 14 QTLs responsible for sesame resistance to charcoal rot disease. More recent results from Liang et al. (2021), Asekova et al. (2021), XU et al. (2021), and Yol et al. (2021) have reported high-density genetic linkage maps of sesame using 1,354 bins, 1,662 SNPs, 424 SNPs, and 782 SNPs, respectively (Figure 4). Furthermore, Li et al. (2021) developed a super-dense genetic linkage map of 17 QTLs of seed coat color using 22,375 marker bins in 13 LGs (Figure 4). These findings demonstrate the rapid growth and use of higher-density genetic linkage maps for different sesame traits. The construction of high-density genetic linkage maps and the discovery of QTLs associated with different traits will contribute to the study of key agronomic traits, map-based cloning grain yield, and yield-related genes as well as the application of MAS to improving sesame genetics.

## Genes and QTLs related to sesame plant architecture

Growth habit, plant height, leaf arrangement, leaf shape, and primary and secondary branches are the most important agronomic traits influencing sesame plant architecture (Stavridou et al., 2021). Liang et al. (2021) identified QTLs associated with relative root length, root length, relative shoot length, and shoot length, which contribute significantly to improving sesame architecture. Studying the genetic basis and variability of those traits could contribute not only to sesame architecture but also to the improvement of other oil crops.

The plant leaf is the main and crucial site of photosynthesis; therefore, leaf angle, size, length, shape, width, and overall morphology directly affect CO_2_ assimilation. Sheng et al. (2021) reported three candidate genes (*SIN_1004874*, *SIN_1004882*, and *SIN_104883*) responsible for leaf growth and development. Although the authors mapped 56 QTLs on nine LGs associated with four leaf-related traits using the composite interval mapping method and 81 polymorphic SSR markers, they were not evenly distributed. However, they revealed that 35 of 56 QTLs were clustered on LG 3 and LG 15. This indicates these two linkage groups, containing more than 35 QTLs related to leaf size, could be the best genomic regions for improving sesame architecture. The authors also detected a stable pleiotropic locus (qLS15-1) with large (27.50%) phenotypic contribution rates for leaf length and width. Hence, the gene and genetic diversity of leaf morphology (leaf length and width) are good potential genetic resources for research on improving sesame architecture and yield.

Plant height affects plant architecture and is directly and positively associated with seed yield; thus, improvements in height also improve oil yield (Mustafa et al., 2015; Wang et al., 2016b). Therefore, the genes controlling plant height play a significant role in sesame architecture and the improvement of seed, oil content, and other yield-related traits. Tall plant height is characterized by an indeterminate growth habit, which has a negative effect on logging and the non-synchronous maturity of capsules (Zhang et al., 2018a). This leads to non-uniform capsule maturity, which results in challenges in mechanized harvesting, time, and an increased number of immature seeds and loss of seeds due to dehiscence of the bottom part of the capsules. Improving plant height and developing sesame genotypes with determinate growth habits would solve these problems. Yadav et al. (2022) also suggested that genes and genotypes associated with determinate growth and monocular stems would be important for mechanized sesame harvesting. Therefore, the identification of such genes, genotypes, QTLs, and other genomic regions of sesame related to sesame architecture is important.

Wang et al. (2016a) mapped 41 QTLs for sesame plant height and related traits. Two main QTLS (qPH_8.2 and qPH_3.3) comprising 350 kb on chromosome 8 and 928 kb on chromosome 3 were associated with sesame plant height. QTL qPH-3.3 was responsible for semi-dwarf sesame plant height, which may play an irreversible role in developing determinate sesame genotypes suitable for synchronous maturity and mechanized farming.

Among the 30 QTLs reported by Wu et al. (2014) as grain yield-related traits, four QTLs in LG6 and LG12 were in a region containing genes associated with plant height (Table 4). The authors also identified 16 QTLs related to first-capsule height (eight QTLs in LG4, 11, and 12) capsule axis length (four QTLs in LG5 and 9), and capsule length (six QTLs in LG3, 4, 7, 8, and 12) (Table 5). These QTLs may play significant roles in improving the first-capsule height, plant height, and capsule-axis length, as well as sesame plant architecture. Likewise, Dossa et al. (2019) reported two main QTLs harboring four genes responsible for stem length that were related to drought tolerance (Table 3). The stem length is also a component of plant architecture: as stem length increases, the plant height also increases. They may be positively associated with plant height. Furthermore, Liang et al. (2021) identified 24 QTLs harboring four traits including relative root length, root length, relative shoot length, and shoot length from 11 chromosomes (Table 4). While these traits and QTLs were related to drought tolerance, they encode for plant architecture traits/genes. As they are responsible for shoot and root length, the QTLs could play a significant role in sesame architecture research and improvement. Hence, those QTLs are additional genetic resources for the further sesame improvement program.

**TABLE 3:** QTLs and linkage groups (LGs) associated with sesame architecture traits.

Traits	Detection method				References
	MCIM		MIM		
	12QTLs	LGs	8QTLs	LGs	
1. Plant height (Qph)					Wu et al. (2014)
	Qph-6	6	Qph-6	12	
	Qph-12	12	Qph-12	12
2. First capsule height (Qfch)				
	Qfch-4	4	Qfch-4	4
	Qfch-11	11	Qfch-11	11
	Qfch-12	12	Qfch-12	12
3. Capsule axis length (Qcal)				
	Qcal-5	5	Qcal-5	5
	Qcal-9	9	Qcal-9	9
4. Capsule length (Qcl)				
	Qcl-3	3	Qcl-12	12
	Qcl-4	4		
	Qcl-7	7		
	Qcl-8	8		
	Qcl-12	12		
Other traits from others source				
5. Stem Length (QtlSL)			Candidate genes	Gene name
	QtlSL4.1	4	SIN_1012134	SiTTM3 Dossa et al. (2019)
	QtlSL8.1	4	SIN_1022782	SINIMIN1
			SIN_1022789	SiSAM
			SIN_1022774	SiGOLS1

**TABLE 4:** Twenty-four shoot and root length QTLs associated with plant architecture.

Traits	Chromosome	QTLs	Flanking markers	References
Relative root length	Chr1	*qRRL1*	c01b052-c01b063	Liang et al. (2021)
	Chr3	*qRRL3-1*	c03b043-c03b055	
	Chr3	*qRRL3-2*	c03b102-c03b113	
	Chr7	*qRRL7*	c07b020-c07b028	
	Chr12	*qRRL12*	c12b032-c12b036	
Relative shoot length	Chr1	*qRSL1-1*	c01b035-c01b049	
	Chr1	*qRSL1-2*	c01b109-c01b113	
	Chr11	*qRSL11*	c11b044-c11b051	
Shoot length	Chr1	*qSLC1*	c01b092-c01b100	
	Chr5	*qSLC5*	c05b087-c05b094	
	Chr8	*qSLC8*	c08b065-c08b070	
	Chr12	*qSLC12*	c12b062-c12b072	
Root length	Chr1	*qRLC1*	c01b086-c01b087	
	Chr4	*qRLC4*	c04b040-c04b046	
	Chr6	*qRLC6*	c06b129-c06b135	
	Chr10	*qRLC10*	c10b076-c10b080	
Shoot length-PEG	Chr1	*qSLP1*	c01b032-c01b035	
	Chr8	*qSLP8*	c08b055-c08b063	
	Chr9	*qSLP9-1*	c09b015-c09b021	
	Chr9	*qSLP9-2*	c09b31-c09b033	
Relative length-PEG	Chr1	*qRLP1*	c01b062-c01b070	
	Chr6	*qRLP6*	c06b054-c06b060	
	Chr7	*qRLP7*	c07b030-c07b036	
	Chr12	*qRLP12*	c12b032-c12b036	

More recently, Berhe et al. (2021) reported 19 QTLs and 26 functional genes related to sesame root length. The nine potential genes responsible for root length were *SIN_1017810*, *SIN_1017811*, *SIN_1017812*, *SIN_1017815*, *SIN_1017818*, *SIN_1017843*, *SIN_1007064*, *SIN_1007065*, and *SIN_1020072*. Root length is one component of plant architecture; thus, these genes may be important in improving sesame architecture. These genes are also considered a resource for sesame research programs.

## Genes, genotypes, and QTLs related to sesame yield

Improving sesame seed production and yield-related traits has received attention in recent years and remains a primary goal of oil seed breeding (Islam et al., 2016). A study in Ethiopia identified four genotypes (Setit-2 (G4), G6, G12, and G13) as a genotype/genetic resource for stable and high-yielding rain-fed and irrigation production (Baraki et al., 2020). Baraki et al. (2020) identified WARC-60 (G4) and the Setit-2 variety as high-yielding genotypes with better performance in rainfed production, while Setit-1 (G6) and WARC-60 (G4) were recommended for irrigation production. Similarly, Hirhir, Kibebew, Airless sel-1, Maru (G12), and Marusel-1 (G13) showed relatively good agronomic performance and should be preserved and used for further sesame breeding. Adu-gyamfi et al. (2019) identified and reported six promising accessions (C3, C4, S5, W1, W3, and W5) from five different clusters with higher capsule numbers and seed/capsule. Therefore, those accessions and genotypes are candidate genetic resources in sesame improvement of yield and yield component traits.

Sabag et al. (2021) also discovered major genomic regions on LG2 with strong correlations with flowering date and yield components, suggesting the crucial role of phenology in sesame production. Wu et al. (2014) also reported 30 QTLs, 10 of which were associated with grain yield, including two QTLs related to the number of capsules per plant, six QTLs related to grain number/capsule, and two QTLs related to thousand-grain weight from the RIL population (Table 5). Similarly, Du et al. (2019) reported three QTLs associated with thousand-grain weight (Table 5). These QTLs were first discovered in the sesame research program and are the main resources for further sesame breeding programs. Consequently, Dossa et al. (2019) also identified one QTL (QtlY4.1) and two candidate genes (*SIN_1012139* and *SIN_1012134*) in LG4 related to seed yield. Although these genes were identified in a study of drought-tolerant genes, they may also be candidate genes for sesame seed yield. Yield is a polygenic trait controlled by many genes with cumulative effects that are also influenced by the environment (Wu et al., 2014). Therefore, special techniques for utilizing the identified gene locations are required, including pyramiding more genes or improving many genes simultaneously *via* new technologies such as gene editing.

**TABLE 5:** QTLs for sesame grain/seed yield and yield-related traits.

Traits	Detection method				References
	MCIM		MIM		
	5QTLs	LGs	5QTLs	LGs	
1. Grain number per capsule (Qgn)					Wu et al. (2014)
	Qgn-1	1	Qph-1	1	
	Qgn-6	6	Qph-6	6	
	Qgn-12	12	Qgn-12	12	
2. Capsule number per plant (Qcn)					
	Qcn11	11	Qcan-11	12	
3. Thousand grain weight (Qtgw)					
	Qtgw -11	11	Qtgw 11	11	
**Other QTLs from others sources**				
4. Thousand seed/grain weight	QTLs	Marker intervals		Du et al. (2019)
	Qtsw4	MK1268296-MK1268983		
	Qtsw9	MK193210-MK167922		
	Qtsw12	MK1695007-MK1754691		
5. Seed yield	QtlY4.1	Candidate genes	Gene names	Dossa et al. (2019)
		*SIN_1012139*	*SiABI4*	
		*SIN_1012134*	*SiTTM3*	

LG: linkage group, QTL: quantitative trait loci.

Therefore, the identified QTLs and genes associated with yield and yield-related traits included seed yield, capsule length, grain/capsule, thousand-grain weight, and capsule number/plant and provide opportunities for improving sesame grain yield.

Wei et al. (2015) discovered two promising candidate genes at the locus of flowering time (*SiDOG1* and *SiIAA14*) and two candidate genes at the locus of plant height (*SiDFL1* and *SiILR1*) which were significantly correlated with oilseed yield. Those genes and locations may contribute to improving sesame seed yield. The identification, mapping, and development of databases for sesame yield-related traits would serve as a primary foundation for the application of marker-assisted selection and improvement of yield and yield-related traits.

## Genes and QTLs associated with sesame quality

### Sesame oil

Sesame is produced for its main product of high-quality oil, which is used for cooking, medicine, and cosmetic applications. More than 65% of global sesame seed production is used for the extraction of edible oil, whereas the rest 35% is used for confectionery purposes (Dagmawi et al., 2015). Sesame seeds provide high-quality oil (45–60%), proteins (18–25%), carbohydrates (3–25%), essential vitamins, minerals, as well as specific antioxidants (sesamin and sesamolin) (Muthulakshmi Chellamuthu and Swaminathan, 2012; Dossa et al., 2017b). The quality of sesame oil is determined by the content of the seed oil and the composition of the fatty acids. Although sesame has lipid-related genes, data from online databases indicate a lower number of lipid-related genes (708) compared to other oil-producing crops, including *A. thaliana* (736), soybean (1,298), tomato (902), grapevine (732), and rice (805) (Wang et al., 2014). This may be due to differences in lipid biosynthesis and genome sizes. Differences in genome size may also result in different numbers of genes involved in oil/lipid biosynthesis.

Plant lipid transfer protein (LTPs) functions by transferring phospholipids and fatty acids between membranes *in vitro* (Song et al., 2021). Fifty-two (52) *S. indicum* lipid transfer proteins (*SiLTP*s)/genes were nonrandomly distributed on 13 chromosomes, 75% of which were located on chromosomes 1, 6, 11, and 3, while no *SiLTP* genes were observed on chromosomes 4, 5, or 7 (Song et al., 2021). *SiLTP* genes are expressed in various parts and tissues of sesame, including the capsule, seeds, flowers, leaves, stems, and root tissues (Choi et al., 2008). However, some *SiLTP* genes are located and expressed in specific tissues; e.g. tandem duplication gene pairs of *SiLTPI.4*, *SiLTPI.5* and *SiLTPI.6* on chromosome 1 are expressed in seeds, suggesting their role in seed development (Song et al., 2021).

**FIGURE 5:**
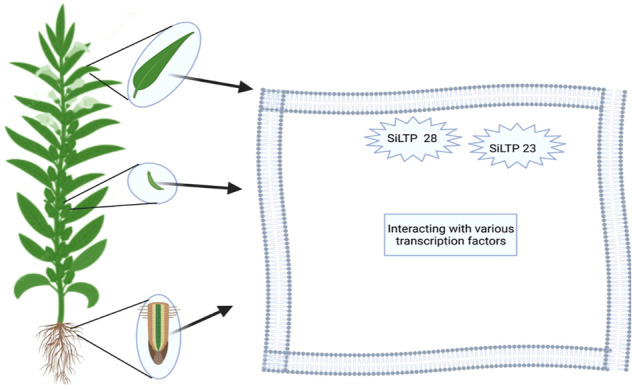
Site of lipid transfer proteins.

Moreover, *SiLTP* genes have multi-function and are expressed in many important agronomic traits. Song and colleagues identified five *SiLTPIs* (*SiLTPI*.10, *SiLTPI*.15, *SiLTPI*.19, *SiLTPI*.26, and *SiLTPI.*2), which were upregulated in all three seed-development stages for high-oil content, while *SiLTPI*.23 and *SiLTPI.28* were candidate genes for high-oil content for the improvement of sesame seeds. These genes showed significantly different expression patterns in sesame growth and development. *SiLTPI*.23 and *SiLTPI.28* showed higher-oil content 30 days after anthesis (DPA), indicating that *SiLTPIs* are more active during seed development than seed maturity (Figure 5). Finally, the authors discovered that the interactions of multiple transcription factors withnh several *SiLTP* genes may alter fatty-acid biosynthesis and oil content in sesame. Wang et al. (2019) also reported 23 candidate genes involved in oil biosynthesis and responsible for the accumulation of oil in sesame. Among those, three lipid transfer protein genes (*SIN_1019175*, *SIN_1019172*, and *SIN_100009*) showed promise for increasing oil content. Hence, lipid transfer protein genes associated with high oil accumulation may be gene resources for sesame and other oil crops.

Breeders should consider not only oil quality (changes in fatty-acid composition) but also oil seed yield when improving oil seed crops (Wei et al., 2015). From 705 sesame cultivars sequenced, 56 important agronomic traits and 546 QTLs associated with oil yield and quality were discovered in four different environments (Wei et al., 2015). Wei et al. also identified candidate causative genes of *SiKASI* (*SIN_1001803*) and *SiKASII* (*SIN_1024652*), which were highly associated with palmitic-acid (C16:0) and palmitoleic-acid (C16:1) concentration. *SiACNA*, *SiDGAT2*, *SiFATA*, *SiFATB*, and *SiSAD* were also identified as candidate genes for fatty-acid composition variation in sesame cultivars Among them, the two major genes *SiKASI* in palmitic acid synthesis and *SiDGAT2* for triacylglycerol synthesis may be responsible for the variation in unsaturated to saturated fat ratios, resulting a good index for healthy dietary consumption (Wei et al., 2015).

Previous studies focused on improving sesame seed yield, disease resistance, and high-oil yield (XU et al., 2021). Quantitative traits such as yield, oil content, quality, and protein content are polygenic, controlled by many genes with cumulative effects, and are influenced by the environment. In many studies, it was difficult to improve the yield and nutritional quality of any crop due to the negative association between the two traits. However, Wei et al. (2015) reported the absence of association (Pearson’s correlation *r* ^2^ = 0.02) between seed yield and oil content of sesame, suggesting the possibility of simultaneous improvement. The gene with the strongest correlation with sesame oil content also showed a strong association with the two lignin compounds, sesamin and sesamolin, that are good for human health and protein content in sesame seeds. Thus, both oil content and sesamin and sesamolin compounds can be improved simultaneously. Two lipase-encoding genes (*CXE17* (*SIN_1003248*) and *GDSL*-like lipase (*SIN_1013005*)), as well as the two lipid transfer protein-encoding genes (*SIN_1019167* and *SIN_1009923*), were identified in four loci encompassing genes encoding for the oil metabolism pathway (Wei et al., 2015). The authors also reported another gene locus containing a candidate gene for oil content (*SiPPO*, *SIN_1016759*) and confirmed that mutations on *SiPPO* (*Sesamum indicum* L*.*) predicted polyphenol-oxidase, which plays a significant role in improving the oil content of sesame.

Similarly, a total of 26 QTLs (16 loci associated with sesamin and 10 loci associated with sesamolin) had a linkage map and were identified using 424 SSR markers (Xu et al., 2021). The assessment of potential genetic variation for sesamin and sesamolin revealed two candidate genes (*SIN_1005755* and *SIN_1005756*) at the same locus in two LGs (LG4 and LG8) based on comparative transcriptome analysis. These results suggested the presence of a single gene with a large effect on the expression of both sesamolin and sesamin, which provides genetic information for the further investigation of the regulation of lignin biosynthesis in sesame. A total of 26 gene locations distributed in eight linkage groups (LG2, 3, 4, 5, 8, 9, 11, and LG13) have been identified.

### Sesame seed coat color

**FIGURE 6:**
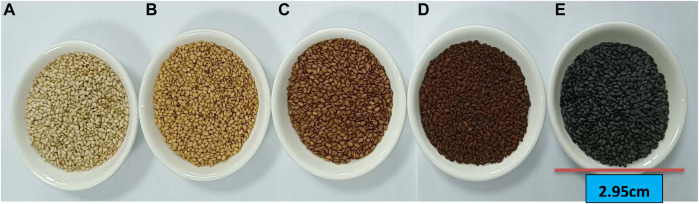
Different seed coat colors of sesame. **(A)** White. **(B)** Yellow. **(C)** Light brown color. **(D)** Brown. **(E)** Black.

Sesame seed coat color is one of the most important agronomic traits that determine sesame quality (Wei et al., 2017). The natural seed coat color of mature seeds of sesame ranges from white, intermediate (grey, golden, brown, yellow, and light white) to black (Zhang et al., 2013b) (Figure 6). Hence, improving the seed coat color and identifying the major and potential candidate genes controlling seed coat color may significantly affect seed quality. Zhang et al. (2013b) identified four gene locations/QTLs (QTL1-1, 11–1, 11–2, and QTL13-1) controlling seed coat color in three linkage groups of sesame, including LG1, LG11, and LG13. Wang et al. (2016b) also identified nine QTLs associated with sesame seed coat color from three LG4, 8, and 11 (Table 7). Similarly, Wu et al. (2014) also reported four QTLs controlling seed coat color with a range of 59-33–69.89% heritability in the F3 population. Subsequently, Wei et al. (2016) identified six candidate genes (*SIN_1016759*, *760*, *761*, *762*, *763*, and *SIN_10120223*) in six QTLs of LG4. However, none of these candidate genes were expressed in qRT-PCR analyses and only one candidate gene *PPO* (*SIN_1016759*), which was responsible for phenol-oxidase and black pigment, was synthesized and expressed in the seeds of the cultivar “Mishuozhima” from 11 to 20 days. Furthermore, Wang et al. (2020) reported 20 candidate genes for seed coat color associated with black pigmentation from the gnome Zhonghzi.33 (Table 6). Among these, 15 candidate genes for seed coat color were annotated whereas five were not.

**TABLE 6:** Twenty candidate genes responsible for seed coat color.

Chromosome	Candidate genes	NCBI annotation
Chr1	*SIN_1013986*	Ferruginol synthase like
Chr2	*SIN_1002392*	Probable carotenoid, cleavage dioxygenase 4, chloroplastic
Chr2	*SIN_1017088*	Not annotated
Chr3	*SIN_1017435*	Not annotated
Chr4	*SIN_1016759*	Polyphenol oxidase I, chloroplastic like
Chr4	*SIN_1006242*	Cytochrome P450 93A3-like
Chr6	*SIN_1018543*	Transcription factor WER-like
Chr6	*SIN_1020696*	Cytochrome P450 71D95-like
Chr6	*SIN_1012414*	Not annotated
Chr7	*SIN_1001138*	Crocetin, glucosyltransferase, chloroplastic-like
Chr8	*SIN_1006025*	Iso-chorismite synthase, chloroplastic
Chr8	*SIN_1026689*	WAT1-related protein At3g28050-like
Chr9	*SIN_1024143*	MLO-like protein 6
Chr9	*SIN_1025570*	Not annotated
Chr10	*SIN_1018917*	Cytochrome P450 71A1-like
Chr10	*SIN_1018959*	Chalcone synthase-like
Chr10	*SIN_1018961*	Chalcone synthase
Chr12	*SIN_1006892*	Uncharacterized protein LOC105175232
Chr12	*SIN_1022200*	Low Quality protein: dihydroflavonol 4-reductase
Chr13	*SIN_1006470*	Not annotated

Data source: Wang et al. (2020).

These genes encode different chemical compounds and contribute to the coloration of the sesame seed coat (Figure 6). Sesame has different genotypes with different seed coat colors, with white-colored and thin seed coat varieties the most favored (Wei et al., 2016; Tripathy et al., 2019). Wei et al. (2016) reported that the cultivated variety “Zhonghzi13” has white seed coat color whereas the landrace “Mishuozhima” has black seed coat color.

Du et al. (2019) identified 14 QTLs in three LGs (LG4, 9, and 12) associated with seed coat color from the F3 population and progeny of a cross between “Gaoyou 8 and Ganzhi 6” (Table 7). Recently, a GWAS study of 366 sesame germplasms in 12 environments performed at Henan Research Centre in China exploring the factors affecting the genetic architecture of seed coat color identified 92 candidate genes associated with seed coat color, which were linked to four SNP markers (Cui et al., 2021). The candidate genes included *SIN_1006005*, *SIN_1006010*, *SIN_1012034*, *SIN_1006006*, *SIN_1006020*, *SIN_1024895*, *SIN_1006022*, *SIN_1016759*, *SIN_1023237*, and *SIN_1023224*. Likewise, Li et al. (2021) more recently identified 17 QTLs/candidate genes for sesame seed coat color in seven non-overlapping intervals on four linkage groups (Table 7). These results showed the clustering of QTLs in a linkage group of nine, which suggested its significance in sesame seed coat color.

**TABLE 7:** Detailed information of QTLs associated with seed coat color in sesame.

Indices	Linkage group (LGs)	Quantitative trait loci (QTL)	References
L	3	qSCL3.1	Li et al. (2021)
	9	qSCL9.1	
	9	qSCL9.2	
	9	qSCL9.3	
	9	qSCL9.4	
	4	qSCL-4.1	Wang et al. (2016b)
	8	qSCL-8.1	
	11	qSCL-11.1	
	4	qsccL*4	Du et al. (2019)
	4	qsccL4	
	4	qsccL4	
A	5	qSCL5.1	Li et al. (2021)
	6	qSCL6.1	
	9	qSCL9.1	
	9	qSCL9.2	
	4	qSCa-4.1	Wang et al. (2016b)
	8	qSCa-8.1	
	8	qSCa-8.2	
	4	qscca*4	Du et al. (2019)
	4	qscca*4-2	
	4	qscca*4-1	
	9	qscca*9	
B	3	qSCb3.1	Li et al. (2021)
	3	qSCb3.2	
	5	qSCb5.1	
	5	qSCb5.2	
	9	qSCb9.1	
	9	qSCb9.2	
	9	qSCb9.3	
	9	qSCb9.4	
	4	qSCb-4.1	Wang et al. (2016b)
	8	qSCb-8.1	
	11	qSCb-11.1	
	4	qsccb*4	Du et al. (2019)
	4	qsccb4	
X	4	qsccX4	
Y	4	qsccY4	
	9	qsccY9	
Z	9	qsccZ9	
	12	qsccZ12	

Here, the three indices, i.e., L = luminosity, a and b represent the shade of color pairs.

## Genes and QTLs associated with disease resistance in sesame

Sesame plants are mainly affected by biotic stress induced by bacteria, fungi, viruses, insect pests, and nematodes (Yadav et al., 2022), which can cause highly reduced sesame quality and yield. The major diseases of sesame include various bacterial, fungal, and viral agents, including *Cercospora sesami*, *Alternaria sesami*, *Cylindosporium sesami*, *Macrophomina Phaseoli*, *Phytophhthora parasitica*, *oidim spp*, *Erysiphe cirhoracearum*, *Phytophthora nicotianae* var. sesame, and *Helminthosporim sesamine* (Min & Toyota, 2019). Soil-born fungal diseases charcoal rot (*Macrophomina phaseolina* (Tassi) Goid (MP) and root rot (*rhizoctonia solani*) are also problems in sesame cultivation (Teklu et al., 2022). Moreover, the viral disease phyllody (*Orosius albicinctus*), which is associated with phytoplasma, is another problem for sesame production that affects most cultivated and wild species of sesame, resulting in economic loss (Yadav et al., 2022). Phyllody had been reported in Pakistan, African countries (Ethiopia, Burkina Faso, Nigeria, Tanzania, and Uganda), some Asian countries, and India, Iraq, Israel, Myanmar, Venezuela, Thailand, Oman, Turkey, and Mexico (Yadav et al., 2022).

Any new trait in a crop could have resulted from the effort of breeders, geneticists, and pathologists. The development of disease- and pest-resistance genes and genotypes in sesame requires serious knowledge and collaboration among these experts. The production and improvement programs for sesame crops through hybridization, selection, and mutation are also challenged by wilt disease (*Fusarium oxyporum* f. sp. *sesame;* Bayoumi & El-Bramawy, 2007) and charcoal rot (*Macrophomina phaseolina* (Tassi) Goid (MP)) (Yan et al., 2021). For many years, due to a lack of molecular-level understanding and investigation, root rot seriously affects sesame yield and quality. Wang et al. (2017) reported a novel genetic map of sesame using 424 SSR markers, in which they discovered 14 novel QTLs related to charcoal rot resistance (Table 8). The three QTLs (qCRRT8.2, 8.3, and qCRR12.2) detected in different environments had a higher phenotypic contribution rate and could be used to identify candidate genes for disease resistance. These genetic data could also be an important genetic resource for marker-assisted sesame improvement to solve charcoal rot disease.

**TABLE 8:** Detailed information on 14 QTLs responsible for the charcoal-rot disease resistance gene in sesame.

QTLs	Linkage group	Position (cM)	Flanking markers	References
*qCRR3.1*	3	24.50	ZMM2997∼ZMM1033	Wang et al. (2017)
*qCRR3.2*	3	39.30	ZMM5636∼ZMM5775	
*qCRR3.3*	3	52.30	ZMM2218∼ZMM4682	
*qCRR3.4*	3	58.40	ZMM4682∼ZMM5444	
*qCRR5.1*	5	116.80	ZMM1155∼ZMM0314	
*qCRR8.1*	8	10.50	ZMM5060∼ZMM5061	
*qCRR8.2*	8	115.70	ID0041∼ZM638	
*qCRR8.3*	8	123.70	ZM638∼ZMM1682	
*qCRR9.1*	9	104.70	ZMM2323∼ZMM0205	
*qCRR12.1*	12	53.80	ID0046∼ID0133	
*qCRR12.2*	12	89.80	ZMM0913∼ZMM3752	
*qCRR12.3*	12	106.10	ZMM3683∼ZMM2365	
*qCRR13.1*	13	43.90	ZMM1307∼ID0030	
*qCRR13.2*	13	73.50	ZMM2344∼ZMM2343	

Although replacing old and obsolete varieties with new and improved varieties with disease resistance is the cheapest, easiest, and most effective method for introducing disease-controlling mechanisms (Gupta et al., 2018); collecting, evaluating, identifying, and conserving the genetic resources with potential disease-resistant traits is a sustainable and a key activity in crop improvement programs. Gupta et al. (2018) reported 18 genotypes/genotype resources related to tolerance for major diseases in sesame in India. JT-21, TKG-21, TKG-22, TKG-55, JTS-8, RT-46, RT-48, RT-54, RT-103, RT-125, RT-127, Sekhar, Guatam, Usha, TSS-6, M-75, Swetha, and Nirmala are resistant to different major diseases of sesame. Therefore, these genotypes could be further used as parental resources in sesame improvement programs.

Comparative transcriptome analyses of resistance to sesame charcoal rot (*Macrophomina phaseolina*) by Yan et al. (2021) identified several core genes, including protein kinase, disease-related proteins, cytochrome P450s and peroxidases, and other closely-related genes. The authors also identified 52 significantly and differentially expressed genes as a response to hormones (ABA and JA), cell-wall, hormone-mediated signaling, cell-cell junction defense response, and signal transduction and hormone-mediated signaling pathway associated with plant stress, especially sesame charcoal rot (*Macrophomina phaseolina*)*.* Similarly, Radadiya et al. (2021) reported 1,153 candidate genes related to disease resistance from genotype (GT-10). Furthermore, Asekova et al. (2021) reported the upregulation of *SIN-1019016* and three promising and potential QTLs (qPhn_10KACC481220, qPhn_10_KACC48121, and qPhn_10_No2526) against Phytophthora blight (PB) caused by *Phytophthora nicotiana*. The upregulation of *SIN-1019016* in the resistant line would contribute to the development of PB resistance. Hence, those QTLs were genomic regions harboring five candidate genes associated with PB in sesame. Although no evidence has been reported to cure phyllody disease due to the overlap of its symptoms with environmental effects, it is possible to develop resistant varieties from interspecific crosses of wild and cultivated species (Yadav et al., 2022). In this regard, Junior et al. (2019) identified two resistant accessions (ACS38 and ACS108) from the screening of 542 sesame genotypes in field and greenhouse conditions. Additional molecular details about the function of this genomic area in regulating PB resistance in sesame may be obtained *via* functional characterization and expression analysis of other genes in the QTL intervals.

## Genes and QTLs associated with abiotic stress tolerance in sesame

### Drought and waterlogging tolerance genes and QTLS

Drought and waterlogging are major abiotic stress factors for limiting sesame production in the early growth stages (Liang et al., 2021; Yadav et al., 2022) and development (Li et al., 2017). Drought tolerance is a key and well-known trait of sesame breeding programs for plant growth and yield stability (Liang et al., 2021). In recent decades, several SNP markers have been employed for QTL genetic mapping and GWAS analysis, thanks to the development of next-generation sequencing (NGS) technology and the availability of the complete genome sequence of sesame (Liang et al., 2021). Based on linkage disequilibrium, the genome-wide association study (GWAS) technique is frequently used to identify associations between molecular markers, candidate genes, and traits of interest in a particular population (Li et al., 2018).

Among the plant-specific transcription factor families, the homeodomain-leucine zipper (HD-Zip) gene family is highly involved in plant growth, development, and various stress responses (Zhang et al., 2021). However, sesame lacks genes responsible for responses to drought and salinity stresses. Zhang et al. (2021) reported 45 HD Zip candidate drought-resistant genes (*SiHDZ01-SiHDZ45*). The 44 *SiHDZ* genes map on the sesame genome in 12 linkage groups. These 45 candidate genes for drought resistance are important for further sesame improvement and are considered new gene resources.

Evidence has also indicated that *WRKY* are involved not only in the response to cold, drought, and heat stresses but also to waterlogging stress. Li et al. (2017) identified 26 candidate *WRKY* and *SiWRKY* genes that may play a role in sesame drought stress responses. Liang et al. (2021) reported that four genomic regions were associated with five significant QTLs (qRSL1.2, qRSL1, qRSL7and qRSL12/qRSL12) based on the respective flanking markers in each drought-resistant gene QTL. Although 465 genes were identified in these four genomic regions, only 347 genes were functionally annotated; the remaining 118 genes were unknown proteins/hypothetical proteins or repetitive. Their study identified and described 34 QTLs responsible for drought tolerance traits in different chromosomes with flanking markers (Table 9). Furthermore, Liang et al. identified twelve chromosome regions linked to relative shoot length (RSL), relative seedling weight (RSW), and relative root length (RRL).

**TABLE 9:** Detailed information on drought tolerance traits and their QTLs.

Traits	Chro. Loca	Locus	Flanking markers	Traits	Chromo. Location	Locus	Flanking markers
Seedling weight control	Chr2	*qSWC2*	c02b067-c02b073	Shoot length-PEG	Chr1	*qSLP1*	c01b032-c01b035
	Chr8	*qSWC8*	c08b086-c08b090		Chr8	*qSLP8*	c08b055-c08b063
	Chr12	*qSWC12*	c12b069-c12b071		Chr9	*qSLP9-1*	c09b015-c09b021
Shoot length control	Chr1	*qSLC1*	c01b092-c01b100		Chr9	*qSLP9-2*	c09b031-c09b033
	Chr5	*qSLC5*	c05b087-c05b094	Root length-PEG	Chr1	*qRLP1*	c01b062-c01b070
	Chr8	*qSLC8*	c08b065-c08b070		Chr6	*qRLP6*	c06b054-c06b060
	Chr12	*qSLC12*	c12b062-c12b072		Chr7	*qRLP7*	c07b030-c07b036
Root length control	Chr1	*qRLC1*	c01b086-c01b087		Chr12	*qRLP12*	c12b032-c12b036
	Chr4	*qRLC4*	c04b040-c04b046	Relative seedling weight	Chr5	*qRSW5-1*	c05b019-c05b029
	Chr6	*qRLC6*	c06b129-c06b135		Chr5	*qRSW5-2*	c05b071-c05b074
	Chr10	*qRLC10*	c10b076-c10b080		Chr6	*qRSW6*	c06b041-c06b045
Seedling weight PEG	Chr1	*qSWP1*	c01b003-c01b010		Chr12	*qRSW12*	c12b061-c12b072
	Chr3	*qSWP3*	c03b116-c03b119	Relative Shoot length	Chr1	*qRSL1-1*	c01b035-c01b049
	Chr9	*qSWP9*	c09b031-c09b040		Chr1	*qRSL1-2*	c01b109-c01b113
Relative root length	Chr1	*qRRL1*	c01b052-c01b063		Chr11	*qRSL11*	c11b044-c11b051
	Chr3	*qRRL3-1*	c03b043-c03b055				
	Chr3	*qRRL3-2*	c03b102-c03b113				
	Chr7	*qRRL7*	c07b020-c07b028				
	Chr12	*qRRL12*	c12b032-c12b036				

Source: Liang et al. (2021), (https://doi.org/10.1371/journal.pone.0247681.t004).

Although more than 300 QTLs and more than 250 candidates have been identified in sesame, QTLs and important genes for chlorophyll yield, heat tolerance, waterlogging tolerance, and other traits are under investigation (Berhe et al., 2021). Different genotypes respond differently to waterlogging stress and the resistant genotypes remain more stable during waterlogging, whereas more sesame genotypes showed sensitivity to waterlogging stress (Wang et al., 2016a). Among the genes expressed in response to waterlogging, 66 candidate genes have been shown to improve sesame tolerance to waterlogging stress (Wang et al., 2016a). These 66 genes cluster in five homologous stress-responsive genes (*SIN_1024017*, *SIN_1021706*, and *SIN_1012279*). Their expression patterns were validated by real time-PCR.

Yanxin et al. (2014) also identified six QTLs linked to water-logging resistance traits (Table 10). Those six QTLs were located on LG7, 9, 13, and 15 and were identified by using the SSR marker (ZM428), with an average linkage distance of 0.7 cM. The author also identified eight germplasms (2413, 2552, Ezhi-1, Funan Zhima, Henan-1, Jiaxing Jinkouhei, Macheng Heizhima, and Xiping Erlanghua) from the sesame core collection showing tolerance to waterlogging. Furthermore, Dossa et al. (2019) discovered ten major and stable QTLs from four LGs (LG4,6,7, and 8) in their genome-wide association study of drought tolerance-related traits from among 400 sesame accessions in a 2-year experiment (Table 11). Though they identified 569 significant SNPs across 10LGs of the sesame genome, 21 potential candidate genes were identified using 10 SNPs from 4LGs for five traits.

**TABLE 10:** QTLs associated with water logging tolerance in sesame.

Name of QTLs	Range	Position	References
1. qEZ09ZCL13	ZM22-ZM92	0.0	Yanxin et al. (2013)
2. qWH09CHL15	E16M19-E14M14a	8.0	
3. qEZ10ZCL07	E5M12a-ZM351	4.5	
4. qWH10ZCL09	M20E10-ZM428	7.0	
5. qEZ10CHL07	E5M12a-ZM351	4.5	
6. qWH10CHL09	M20E10-ZM428	7.0	

**TABLE 11:** Identified drought-tolerant QTLs, genes, and SNPs in sesame.

Traits	LG	QTLs	Candidate gene ID	Gene name	References
Capsule number	8	QtlCN8.1	*SIN_1022782*	*SiINIMIN1*	Dossa et al. (2019)
			*SIN_1022789*	*SISAM*	
			*SIN_1022774*	*SiGOLS1*	
Stem Length	4	QtlSL4.1	*SIN_1012134*	*SiTTM3*	
	8	QtlSL8.1	*SIN_1022782*	*SINIMIN1*	
			*SIN_1022789*	*SiSAM*	
			*SIN_1022774*	*SiGOLS1*	
Survival rate	4	QtlSR4.2	*SIN_1012139*	*SiAB14*	
			*SIN_1012134*	*SiTTM3*	
	6	QtlSR6.1	*SIN_1015691*	*SiP450*	
		QtlSR6.2	*SIN_1015693*	NA	
			*SIN_1005662*	*SiPOD3*	
	7	QtlSR7.1	*SIN_1004723*	NA	
			*SIN_1004716*	NA	
	8	QtlSR8.1	*SIN_1022782*	*SiNIMIN1*	
			*SIN_1022789*	*SiSAM*	
			*SIN_1022774*	*SiGOLS1*	
Wilting level	7	QtlWL7.1	*SIN_1004723*	NA	
			*SIN_1004716*	NA	
Seed yield	4	QtlY4.1	*SIN_1012139*	*SiABI4*	
			*SIN_1012134*	*SiTTM3*	

### Genes and genotypes and QTLs associated with salt tolerance in sesame

Soil salinity is a serious and major problem that affects the production and productivity of crop plants including sesame (Zhang et al., 2019; Raza et al., 2022). Several studies have identified candidate genes, QTLs, promising genotypes, and genomic regions associated with salt tolerance (Lakhanpaul et al., 2012; Suassuna et al., 2017; Zhang et al., 2019). Suassuna et al. (2017) identified two promising genotypes (LAG-927561 and LAG-26514) which showed moderate resistance to salt stress and adaptation. Similarly, Zhang et al. (2019) reported 59 salt-tolerant/upregulated candidate genes from two sesame accessions, salt-tolerant (WZM3063 (ST)) and salt-sensitive (ZZM4028 (SS)) obtained through the evaluation of salt tolerance in 490 sesame core collections stored in the China National Gene Bank at the Oil Crops Research Institute Among these, ABC transporter (*LOC-105170264*), β-glucoside (*LOC_105173929*), cytochrome P450 (*LOC_105161642*), dehydration-responsive element-binding protein (*LOC_105157670*), and UDP-glycosyltransferase (*LOC_105171082*) genes of regulatory elements were identified.

Despite the differences in tolerance levels, 901 genes (206up-regulated and 695down-regulated) were constitutively expressed in both genotypes and constituted the core genes linked to salt stress response (Zhang et al., 2018b). In this regard, 101 transcription factors from 31 gene families were discovered, including *AP2-EREBP*, *bHLH*, *bZIP*, *HB*, *MYB*, and *NAC.* Their different expression patterns indicated their essential regulatory role in response to salt stress. Under salt stress, *PYLs*, *PP2Cs*, *SnRK2s*, *AREB/ABFs*, *VP1*, and *LEA* were activated or repressed, implying their critical roles in ABA signal transduction and protecting sesame from salt stress-induced damage.

## Other genes and QTLs

### Gene resources and QTLs associated with non-shattering traits

Seed yield reduction in sesame occurs due to high capsule shattering at harvest and uneven capsule ripening characteristics of the sesame crop (Abdellatef et al., 2008; Yol & Uzun, 2019; Teklu et al., 2022). The scientific evidence indicates that capsule shattering can lead to up to a 50% yield loss during or after harvesting in sesame (Phumichai, et al., 2017). Besides yield reduction, the absence of noon shattering/indehiscent cultivars suited for mechanical harvesting is another topic for sesame improvement (Abdellatef et al., 2008). Therefore, developing low-shattering or shattering-resistant varieties of sesame could improve yield and allow mechanized farming techniques (Tripathy et al., 2019; Yadav et al., 2022). Developing these varieties requires variability among sesame genotypes worldwide or the identification of a gene or genetic resources for the improvement of sesame shattering traits (Qureshi et al., 2022). In Bulgaria, four sesame varieties (victoria, aida, valya, and nevena) have been developed that are suitable for mechanized farming (Myint et al., 2020). Although the shattering trait is considered to be qualitative and controlled by a single major gene (Yol et al., 2021), this topic has received less attention, and research to identify indehiscent/shattering resistant candidate genes for sesame is required.

Wongyal et al. (1997) aimed to develop shattering-resistant genotypes using gamma rays and ethyl methanesulfonate (EMS)-induced mutations in the Kasetsart University Sesame Breeding Project in Thailand. Five promising mutant lines (M-6060 to M-6064) from the gamma-ray treatment M-6045, M-6015, and M-6054 lines with 1.0% EMS mutagen treatments were obtained. These lines could serve as a source of shatter-resistant genes for further sesame breeding work.

Although the method of harvesting matters, the first gene/genotypes with capsule shattering resistance/indehiscence (homozygous recessive allele, *id*/*id*) was identified in1943 by Langham in Venezuela through successive mutation and published in 1946 (Langham, 2001). Later, Wongyai et al. (1997) and Diouf et al. (2010), reported different mutants of delayed, closed capsule, and semi-shattering (Saha & Paul, 2017). Similarly, 52 mutants were identified with shatter resistance in both Tillotoma and Rama cultivars in West Bengal (Saha & Paul, 2017). The capsule varieties with the recessive *idid* allele showed a strengthened zone of weakness due to the presence of numerous cell layers between the epicarp and median vascular bundle, with no band of small thin-walled cells in this region (Day, 2000).

In addition, Zhang et al. (2018) discovered the candidate gene *SiCL1* in sesame, which controls leaf curling and capsule indehiscence. The authors reported that *SiCL1* is mainly expressed in the tissues of leave, stems, buds, and capsules. Hence, identifying the gene and localization plays a role in the improvement of sesame capsule indehiscence, which complicates mechanized farming. However, Yol et al. (2021) suggested improving this trait using gene editing tools instead of agronomic improvement approaches because of the reduced suitability of lower-yielding genotypes for mechanized harvesting. The genes, gene locations, and genetic materials associated with indehiscence traits in sesame can be used as resources for the future improvement of this trait in sesame research programs.

### Candidate genes and QTLs related to mineral nutrients

Although 70% of sesame is processed into cooking oil (Sonia et al., 2015), it is also a good source of carbohydrates, proteins, fat, and several mineral nutrients (Makinde and Akinoso, 2013). Some evidence also supports sesame seeds and oil as sources of vitamin E, which has antioxidant properties and is important for lowering blood cholesterol levels (Kamal et al., 2013; Tripathy et al., 2019). Teboul et al. (2020) identified the candidate genes and QTLs associated with sesame seed mineral nutrient concentration. Among these were 381 potential candidate genes, 285 of which were not annotated and 96 of which were uncategorized. However, 36 candidate genes in three LGs (8, 11, and 16) were annotated (Table 12). Among the QTLs identified, QTL-qk-1 in LG8 showed two candidate genes: SKOR-like (*LOC-105167760*) and SKOR-potassium channel (*LOC-105167785*) Similarly, in three QTLs (qZN_5 and qFe_6, qS_2), *LG11*, phosphate starvation response 1-like protein; *PHR1* (*LOC-105173373*), which is responsible for seed zinc and iron concentration, and three genes encoding a cyclic nucleotide-gated ion channel-1 like (*CNGC1*) (*LOC-105173138*, *LOC-105173087*, and *LOC-105173088*) were identified. Besides, the zinc transporter 8 (*LOC-105178590*) and zinc transporter 8-like (*LOC-105178589*) genes, which determine the zinc content of sesame seeds, were identified in LG6 and the QTL of qZn-6.

**TABLE 12:** Candidate genes and their protein annotation for mineral nutrients.

LG	Marker interval	Traits	Candidate gene ID	Annotated protein’s function
LG8	4550182–7125811	FD, FN, NN, IL, SB, CL, CDI, SL, TSW, SC, Zn, Fe, Cu, Mn, Ca, Mg, K	*LOC105167760*	Potassium channel SKOR- like
			*LOC105167785*	Potassium channel SKOR
			*LOC105167762*	Inositol hexakisphosphate and di-phosphoinositol-pentakisphosphate kinase 2-like
			*LOC105167788*	Ethylene-responsive transcription factor 1B-like
			*LOC105167789*	Ethylene-responsive transcription factor 1B-like
			*LOC105167791*	Ethylene-responsive transcription factor 1B-like
			*LOC105167765*	Transcription repressor KAN1
			*LOC105167815*	Isocitrate dehydrogenase [NADP]
LG11	310665–1216709	NN, CDI, SL, SW, SP, SA, Zn, Fe, S	*LOC110012885*	Ethylene-responsive transcription factor ERF023-like
			*LOC105173138*	Cyclic nucleotide-gated ion channel 1-like
			*LOC105173087*	Cyclic nucleotide-gated ion channel 1-like
			*LOC105173088*	Cyclic nucleotide-gated ion channel 1-like
			*LOC105173140*	MYB-like transcription factor ETC3
			*LOC105173141*	Probable WRKY transcription factor 30
			*LOC105173122*	Heavy metal-associated isoprenylated plant
			*LOC105173253*	Probable polygalacturonate protein 39-like
			*LOC105173155*	Ferric reduction oxidase 2
			*LOC105173156*	Ferric reduction oxidase 2-like
			*LOC105173161*	Ascorbate transporter
LG11	5566003–5767772	NN, IL, SB, PH, CDI, Fe, Zn	*LOC105173373*	Protein phosphate starvation response 1-like
			*LOC105173380*	Citrate synthase
LG11	14205927–14426041	NN, SB, PH, CL, CW, SL, P	*LOC105174482*	Transcription factor LHW
			*LOC105174515*	Transcription factor MYB1
LG16	14816–3048510	CDI, Zn, Mn	*LOC105178592*	Transcription factor bHLH30-like
			*LOC105178450*	Transcription factor TCP10-like
			*LOC105178476*	Nicotianamine aminotransferase A
			*LOC105178598*	WRKY transcription factor 6
			*LOC105178495*	Transcription factor MYB101
			*LOC105178506*	MYB-like transcription factor ETC1
			*LOC105178507*	Isocitrate lyase
			*LOC105178516*	Aconitate hydratase
			*LOC105178613*	Calcium-binding protein PBP1-like
			*LOC105178537*	Transcription factor MYB39-like
			*LOC105178559*	Ethylene-responsive transcription factor 4-like
			*LOC105178589*	Zinc transporter 8-like
			*LOC105178590*	Zinc transporter 8

Source: Teboul et al. (2020).

Teboul et al. (2020) suggested that identifying candidate genes that alter seed mineral-nutrient and morphological traits can serve as a good foundation for future sesame research integrating transcriptional expression, allele mining, and fine mapping of potential candidate genes.

A GWAS study conducted by He et al. (2019) in Kongju National University, Korea (2015) using 92 sesame accessions reported two candidate QTLs: (*LG08_6621957* (A/G) linked to γ-tocopherol content and *LG03_13104062* (C/T) SNP linked to β-tocotrienol) from LG8 and 3, respectively. These two loci may be important for improving the vitamin E content in sesame. He et al. also identified twelve candidate genes responsible for different biomolecules/enzymes related to vitamin E. Among the twelve candidate genes, five (*SIN_1001936*, *SIN_1001937*, *SIN_1001938*, *SIN_1001939* and *SIN_1001940*) were located in LG 8 and seven (*SIN_1022039- up to SIN_1022045*) in LG3. Although several genes related to mineral nutrients were identified, *SIN_1022045* was annotated as a zinc-ion binding protein that regulates ARF-GTPase activity and was associated with the endocytosis process pathway.

## Conclusion

Sesame is a well-studied crop, with an updated database providing its genomic, proteomic, and all other genetic data. The development of the Sinbase platform, which stores the functional genes online and provides free access to genetic and genomic information, enables researchers, experts, and any data-gathering bodies to access this information from anywhere. This is a great scientific contribution to the field related to sesame production. This crop has special features that make sesame oil and its products preferable for consumers due to its higher quality oil and antioxidant properties. Improving sesame agronomic by enhancing important traits and productivity requires more gene and genetic resources with higher diversity. The improvement and research programs for sesame have identified and applied molecular markers including SSR (EST-SSR, gSSR, cSSR), SLAF, RFLP, RAPD, InDels, and others.

Many scientific research results are published online to report useful genetic information obtained worldwide. The present review pooled the genes and genetics resources useful for sesame improvement. Moreover, the genes, QTLs, and genotypes identified in sesame breeding associated with important agronomic traits including seed yield and yield component traits, sesame architecture, seed coat color, disease resistance, drought tolerance, waterlogging resistance, salt tolerance, oil content, quality, and other agronomic traits were presented. The gene and genetic resources identified for sesame improvements will likely play major roles in closely related oil crop species (for example, sunflower, rape seed, etc.), providing an opportunity to identify genes with shared functions.

Although different genetic and genomic data of Sesame are available in Sinbase 2.0 and other online resources, it lacks proper data handling, maintenance, and utilization in places with technology to allow its access, as well as extension to developing countries where higher genetic diversity is present. More than ten online comprehensive sesame genomic and five marker databases are also available. Despite being considered an “orphan crop”, sesame has received large investments in terms of money, time, labor, and effort. Sesame grows relatively quickly to provide higher yields with limited water requirements, lower costs, and good prices globally. Therefore, this crop requires increased attention for production in developed and developing countries to benefit from its beneficial features. This detailed review of sesame provides more knowledge and guiding examples for continuing genetic investigations in sesame as well as other oil crop species with more complex genomes.

## Future prospects

Sesame research is interesting and much progress has been made in improving knowledge of overall important traits, QTLs, and genes as well developing sesame databases. Many genes, QTLs, and other genetic information related to sesame are now available in an online database. However, work is limited regarding the improvement of minerals and vitamins in sesame, as well as sesame architecture including marker development, genes, genomic regions, and QTL identification. Therefore, additional research is required on these topics. It is better to concentrate on protein-protein interactions in the adaptive response of sesame to drought, water-logging, salt stress, and other abiotic stresses and to investigate the genetic basis for the natural variations in sesame tolerance to salinity, drought, water-logging, and other stresses. Future studies should also concentrate on the availability of candidate genes, genotypes, QTLs, and genomic regions of sesame in developing countries with limited production technologies and research resources.

The omics and gene editing era, has allowed the incorporation of useful candidate genes into a single sesame genotype, as well as the development of sesame genotypes characterized by higher yield, higher oil content, quality, and resistance to different stresses. Global efforts in research and development have been diverted to multi-omics approaches including metabolome, transcriptome, and proteome profiling and the investigation of new genetic components useful to perform different functions. Hence, the sesame crop requires more work on its transcriptomics, metabolomics, and proteomics, as well as the integration of all other omics approaches. Although various sesame genetic resources are available in different national and international gene banks, the reports of various scholars may not solve the double counting of germplasms and the lack of general availability and accessibility. Therefore, the establishment of online databases and pangenomes is needed to easily record all genetic resources globally, reduce the double counting of sesame materials, and provide important information on genetic resources.

Generally, sesame research should focus on the following critical research areas:

1. Sesame architecture (root and shoot); i.e., the development of varieties with determinate growth habits and indehiscence capsules suitable for mechanized farming.
2. Online databases (pangenome) that provide accessible data on candidate genotypes, genes, QTLs, and other genomic regions.
3. Multi-omics and gene editing approaches for better trait development in sesame improvement program
4. Improvement of sesame mineral and vitamin content
5. Breeding in the context of biotic and abiotic stress
